# Human CAR Tregs Targeting SOD1 and Expressing BDNF Reduce Inflammation and Delay Disease in G93A hSOD1-NSG Mice

**DOI:** 10.3390/cells14171318

**Published:** 2025-08-26

**Authors:** David J. Graber, W. James Cook, Marie-Louise Sentman, Joana M. Murad-Mabaera, Elijah W. Stommel, Charles L. Sentman

**Affiliations:** 1 Department of Microbiology and Immunology, Geisel School of Medicine at Dartmouth, Lebanon, NH 03756, USA; 2 Center for Synthetic Immunity, Geisel School of Medicine at Dartmouth, Lebanon, NH 03756, USA; 3 Celdara Medical LLC, 16 Cavendish Court, Lebanon, NH 03766, USA; jm@celdaramedical.com; 4 Department of Neurology, Dartmouth-Hitchcock Medical Center, Lebanon, NH 03756, USA; elijah.w.stommel@hitchcock.org

**Keywords:** amyotrophic lateral sclerosis, regulatory T cell, IL-10, inflammation, cell therapy, chimeric antigen receptor

## Abstract

Regulatory T cells (Tregs) have anti-inflammatory immunomodulatory activity and hold therapeutic potential for chronic neuroinflammatory neurodegenerative diseases, such as amyotrophic lateral sclerosis (ALS). We are developing engineered human Tregs with enhanced disease-modifying activity for treating ALS. A combination of a disease-specific chimeric antigen receptor (CAR) recognizing misfolded human superoxide dismutase-1 (hSOD1) and constitutive expression of brain-derived neurotrophic factor (BDNF) was tested. The scFv region of CAR demonstrated binding to anterior horn tissues of ALS patients with and without familial ALS mutations in SOD1. Tregs transduced to express BDNF showed the ability to secrete BDNF and protect co-cultured neuronal cells from peroxidase toxicity. Co-expression of BDNF did not inhibit CAR Treg expansion, Treg markers, or CAR-mediated anti-inflammatory cytokine production. Human Tregs co-expressing CAR and BDNF were tested for activity in G93A hSOD1-NSG transgenic mice, which develop an early-onset and aggressive ALS-like disease and do not reject human cells. Human Tregs expressing CAR and BDNF delayed the onset of disease development, extended survival, and decreased spinal cord neuroinflammation. The engineered Tregs showed enhanced disease-modifying activity and hold promise as a therapy for ALS.

## 1. Introduction

Amyotrophic lateral sclerosis (ALS) is a fatal neurodegenerative disease of progressive motor neuron loss in the central nervous system (CNS) that leads to voluntary muscle weakness and eventual paralysis. Approximately 8% of cases occur as an inherited familial ALS (fALS) form, while most cases are of the sporadic ALS (sALS) form [1]. Activated microglia—resident CNS macrophages—and astrocytes, accumulation of aberrant proteins, and free-radical damage are key features at sites of motor neuron degeneration [2,3,4]. Currently there are no therapies to halt this devastating disease, which has an average survival of two to five years after diagnosis.

Distinct mutations in superoxide dismutase-1 (SOD1) have been identified that cause ALS, which represent 20% of fALS cases. While the main function of SOD1 is to detoxify superoxide anions, point mutations lead to protein misfolding and a toxic gain of function [5]. Misfolding causes SOD1 to revert from its normal dimer formation, and then these aberrant misfolded monomers bind to each other, forming aggregates, and can get shuttled to the extracellular space [6,7,8]. Misfolded SOD1 has been detected in fALS with SOD1 mutations as well as in sALS and fALS without SOD1 mutations [9,10,11]. Misfolded SOD1 in ALS without SOD1 mutations is controversial, with others reporting no misfolded SOD1 using different antibodies and immunochemistry protocols [12].

Higher numbers of regulatory T cells (Tregs) in the blood of ALS patients are associated with slower disease progression [13,14]. Adoptive transfer of mouse Tregs into G93A hSOD1 transgenic mice attenuates disease development [14,15]. The main disease-modifying activity of Tregs is thought to be modulation of microglia-mediated neuroinflammation. While conventional T cells can promote inflammation, Tregs have been shown to have direct anti-inflammatory activity on microglia and macrophages [16,17,18].

Brain-derived neurotrophic factor (BDNF) is neuroprotective to motor neurons. While it has been explored as an ALS therapy, delivery into the CNS has been a challenge [19,20]. Circulating blood Tregs can traverse to enter the CNS [21,22]. BDNF is known to be expressed by some Tregs, and BDNF-positive Tregs have been associated with better outcomes in stroke patients [23].

Therapies that increase Treg numbers or infuse ex vivo expanded Tregs are currently being tested in ALS patients [24,25,26]. While these polyclonal Tregs hold promise, we are developing Tregs with enhanced disease-modifying activity. Human Tregs are amenable to being expanded in vitro and transduced to express chimeric antigen receptors (CARs) that bind to misfolded SOD1 and activate the Tregs to enact their anti-inflammatory activity in vitro [17]. In the current work, we examined the co-expression of disease-site targeting anti-misfolded human SOD1 CARs with the co-expression of BDNF in human Tregs in both models of neuroprotection and neuroinflammation, including in a unique transgenic ALS mouse model, mSOD1-NSG mice, that expresses G93A hSOD1 transgene on an NSG genetic background, develops ALS-like disease, and does not reject injected human Tregs, so it is possible to directly test human therapeutic cells in this model [27].

## 2. Materials and Methods

### 2.1. G93A hSOD1 Transgeninc NSG Mice

Mouse experiments and procedures were ethically conducted under the approval of Dartmouth College’s Institution Animal Care and Use Committee. Mice were bred and colonies maintained at Dartmouth’s Center for Comparative Medicine and Research facility. mSOD1-NSG mice (G93A hSOD1-NSG transgenic mice) were derived by breeding SOD1G93A B6 mice (stock #004435) to female NOD.Cg-Prkdc scid IL2rγ tm1Wjl (NSG) mice and backcrossing to NSG mice [27]. The strain was maintained by breeding mSOD1-NSG mice with NSG mice. Mice used in this study were backcrossed for eight or more generations. For genotyping, tail tissue samples were collected after weaning and used to determine human SOD1 genotype (PCR) and transgene copy number (real-time qPCR) according to the Jackson Laboratory (Bar Harbor, ME) using their recommended primers and procedures. Copy number controls of DNA from an initial hSOD1G93A B6 mouse (positive) and NSG mice (negative) were included in each assay. Mice with a low transgene copy number, a ΔCt (Ct-hSOD1 − Ct-ApoB) that was 0.7 greater than the positive control, were excluded as breeders. mSOD1-NSG mice were monitored for weight and assessed for limb paralysis three times per week starting at seven weeks of age. Peak weight was determined using a moving average of weight over a seven-day span. Mice that lost greater than 15% of peak weight were considered to be at the disease endpoint and euthanized.

### 2.2. Human ALS and G93A hSOD1 Transgeninc Mouse Spinal Cord Samples

The postmortem tissue core spinal cord samples located at Temple University and provided by Target ALS were transferred to Celdara Medical LLC (Lebanon, NH, USA) for the performance of this study. All samples were de-identified and tissues pre-sectioned prior to shipment. Spinal cord samples from 5 familial ALS donors with confirmed mutation of SOD1 and 21 ALS donors without SOD1 mutation (18 sporadic ALS and 3 C9orf72 fALS) were fixed in 10% neutral buffered formalin, and 4 μm paraffin sections were cut for immunohistochemistry (IHC) staining. Sections from 3 non-ALS donors were procured from a commercial source (Biochain, T2234234, Newark, CA, USA).

Spinal cords from a G93A hSOD1 transgenic NSG mouse at disease end-stage (15% weight loss and limb paralysis) and an age-matched non-transgenic NSG littermate were harvested, tissue was submerged in 10% neutral buffered formalin overnight, and submitted to the Pathology Core facility at the Geisel School of Medicine at Dartmouth for paraffin-embedded blocks.

### 2.3. Immunohistochemistry (IHC) and Image Analysis

All sections were stained by the Pathology Core facility at the Geisel School of Medicine at Dartmouth. IHC staining was performed on the Leica Bond RX platform using Leica Bond Polymer Refine Detection Kit (Leica Biosystems, DS9800, Richmond, IL, USA) with DAB chromogen and hematoxylin counterstain according to manufacturer’s protocols (Leica Biosystems). Misfolded SOD1 scFv with a rabbit Fc was used as the primary antibody at 38 ng/mL as described [17]. No antigen retrieval steps were used. Stained sections were scanned at 20× magnification by the Leica Aperio ScanScope system (Leica Biosystems, Richmond, IL, USA).

Scanned digital svs files were exported from the Leica Aperio ScanScope, and images of the anterior horn regions were captured using the QuPath image analysis software v 0.2.0-m2 [28]. The anterior horn regions that were of good quality (minimal tissue damage or non-specific staining) were chosen for quantification. Qupath was used to decouple DAB from the hematoxylin counterstain. Decoupled DAB-stained images were quantified in gray scale using ImageJ 1.53e software (https://imagej.nih.gov/ij) with identical threshold and measurement criteria applied to all samples, and the proportion of positive misfolded SOD1 scFv antibody staining was calculated for the stained area in the anterior horn region.

### 2.4. Expansion and Retroviral Transduction of Human Tregs

The protocol for enriching, sorting, expanding, and transducing human Tregs has been described in detail [17]. In brief, human CD4+ cells were purified from human PBMCs obtained from plateletpheresis using a human CD4+ cell negative selection RosetteSep procedure as directed by the manufacturer (Stem Cell #15022, Vancouver, BC, Canada) and stored frozen in liquid N_2_. CD4+ T cells were thawed in cold XVIVO-15 media and then rinsed in cold MojoSort Buffer (Biolegend 480017, San Diego, CA, USA). Cells were labeled with anti-CD25-PE (1/50, Biolegend 356103) and then with anti-PE magnetic beads (Miltenyi 130-105-639, Bergisch Gladbach, Germany), followed by selection over an MS column (Miltenyi 130-042-401). CD25-enriched CD4 T cells were stained with Zombie UV (Biolegend 423107) in PBS and then CD4-FITC (1/100, Biolegend 317408) and CD127-APC (1/50 Biolegend 351316). Purified human Tregs were isolated by cell sorting based on Zombie UV neg., CD4+, CD25hi, and CD127lo cells using a FACSAria-II cell sorter (Franklin Lakes, NJ, USA). After sorting, cells were counted and stimulated with CD3/CD28 antibody complexes (StemCell 10971) in XVIVO-15 media with 10% heat-inactivated human AB serum (Sigma H3667, St. Louis, MO, USA) and 500 IU/mL human IL-2. After 9 days, cells were restimulated with CD3/CD28 antibody complexes and then spin-transduced with PG13 retrovirus on Retronectin-coated 24-well non-tissue culture plates on days 10 and 11. Cells were returned to XVIVO-15 with human sera and IL2 on day 12 and expanded until day 17. Cells on day 17 were used and characterized immediately or stored frozen in liquid N_2_ for later use.

### 2.5. Retroviral Vector Production

All retroviruses were constructed on a pSFG backbone. Retroviral CAR vector encoding the anti-SOD1 CAR was made by fusing scFv sequences to gene fragments from CD28 hinge, transmembrane, and costimulation and CD3ζ signaling domain as described [17]. Retroviral BDNF vector consisted of the human BDNF gene (NCBI GenBank ref P23560). Retroviral catalase vector, used as a positive control in the hydrogen peroxide toxicity assay, consisted of the human catalase gene (NCBI GenBank ref P04040). Amino acid sequences were converted to DNA using IDT’s online codon optimization tool (www.idtdna.com/CodonOpt, Commercial Park, Coralville, IA, USA), which also removes unwanted restriction sites and hairpin regions in the mRNA. Restriction sites used for cloning were added to the 3′ and 5′ ends. CAR alone, BDNF alone, and catalase vectors each co-expressed a non-active truncated mouse CD19 gene separated by a T2A self-cleaving peptide. Retroviral CAR+BDNF vector consisted of CAR and BDNF separated by a T2A self-cleaving peptide. Gene blocks were then supplied by IDT. Ecotropic MuLV stocks were generated by co-transfecting the retroviral vector and pPsiEco (Clontech 53460, Mountain View, CA, USA) into 293T cells. The 293T virus supernatants were harvested two days after transfection and filtered through a 0.45 μm filter (Millex SLHVR33RS, Duluth, GA, USA) before immediate use or storing at −80 °C. These ecotropic viruses were used to transduce PG13 (GALV envelope) packaging cells to generate virus to infect human cells. PG13-packaged virus supernatants were harvested from confluent cultures and filtered through a 0.45 μm filter, and stored at −80 °C. Virus-producing cell lines 293T and PG13 were cultured in complete DMEM with a high glucose concentration (Cytiva SH30022, Marlborough, MA, USA) supplemented with 10% heat-inactivated FBS (GE HyClone SH30910, Logan, UT, USA), 100 U/mL penicillin/100 mg/mL streptomycin (Hyclone SV30010, Logan, UT, USA), 1 mmol/L sodium pyruvate (Corning Cellgro 25000-CI), 10 mmol/L HEPES (Corning Cellgro 25060-CI, Corning, NY, USA), 0.1 mmol/L MEM non-essential amino acids (Corning Cellgro 25025-CI), and 50 μmol/L 2-mercaptoethanol (Sigma M3148).

### 2.6. Peroxide Toxicity in Neuronal SH-SY5Y Cells

The neuronal cell line SH-SY5Y was obtained from ATCC. A luciferase-expressing SH-SY5Y (Luc-SH-SY5Y) was generated by transduction using retroviral vectors containing a firefly luciferase gene as described [29,30]. Luc-SH-SY5Y neuronal cells were expanded in complete DMEM consisting of 10% heat-inactivated FBS, MEM non-essential amino acid solution, HEPES (10 mM), sodium pyruvate (1 mM), 2-merceptoethanol (50 μM), and penicillin/streptomycin solution and split when reached 60% to 90% confluency. Luc-SH-SY5Y cells were harvested by trypsinization, pelleted by centrifugation at 500× *g* for 5 min, resuspended in incomplete DMEM (without sodium pyruvate and 2-merceptethanol), and cultured in 96-wells. Luc-SH-SY5Y cells at 10,000 cells/0.1 mL/well were plated in tissue culture-treated white luminometer plates (Corning^®^ 3610, Corning, NY, USA). After allowing the cells to adhere for 3 h, transduced Tregs in incomplete DMEM medium were added to Luc-SH-SY5Y cells at 100,000 Tregs/0.05 mL/well (final volume 0.15 mL/well). Co-cultured cells were incubated for 24 h before hydrogen peroxide (H_2_O_2_) was added in 50 μL of incomplete DMEM media at a final concentration of 80 μM, and co-cultures were incubated for 24 h. Luciferin (50 μg/mL) was added to each well, incubated for 30 min, and bioluminescence was measured on a Centro LB 960 microplate luminometer (Bad Wildbad, Germany) (2-s exposure). The relative number of surviving luc-SH-SY5Y neurons was determined by bioluminescence.

### 2.7. Treatment with Human Tregs and Disease Evaluation in mSOD1-NSG Mice

Transgenic mSOD1-NSG and non-transgenic littermates (NTLs) mice were maintained and monitored weekly up to 7 weeks of age. Transduced human Tregs that were expanded for 17 days were rinsed twice in PBS and resuspended in an injection solution consisting of XVIVO-15 media with IL2 (10,000 IU/mL) without serum. When mice reached seven-to-eight weeks of age, Tregs or vehicle were injected IV at 7–20 × 10^6^ cells per mouse in a volume of 0.4 mL Mice were injected with IL-2 in 0.4 mL PBS IP at two (40,000 IU/mouse), four (10,000 IU/mouse), and six (10,000 IU/mouse) days after injection of Tregs. Vehicle-injected mice also received IL-2 IP injections. Treatment groups were mixed within cages, and male and female mice were balanced between groups. For survival experiments, mice were weighed three times per week until mice reached a disease endpoint of 15% loss of peak body weight. Mice were also monitored for paw grip weakness or limping by a scientist blinded to treatments. For experiments measuring inflammatory markers in the spinal cord, mice were weighed and monitored three times per week and euthanized at 13 weeks of age.

### 2.8. Isolation of RNA and Real-Time qPCR of cDNA

Spinal cords were stored in RNA Protect (Qiagen, Germantown, MD, USA) overnight at 4 °C and then stored longer term at −80 °C. Mouse spinal cords frozen in RNAprotect (Qiagen 76104) were thawed on ice, removed from solution, and dried on clean tissues before processing. RNA was purified using a modified procedure incorporating initial Trizol (Invitrogen 15596026, Waltham, MA, USA) purification, followed by secondary purification over RNeasy columns (Qiagen 74104) as per manufacturer’s protocols. The eluted RNA was quantified by spectrophotometry (NanoDrop™ 2000/2000c Spectrophotometer, Thermo Scientific, Waltham, MA, USA). One μg of total RNA was converted to cDNA using a cDNA synthesis kit (Quanta cDNA synthesis 84657, Houston, TX, USA) following the manufacturer’s protocol. Two μL of the resulting cDNA was used in qPCR analyses using a SYBRgreen kit according to the manufacturer (Quanta Perfecta 84069) on a BioRad CTX96 instrument (Hercules, CA, USA). Primers were designed using a web-based program (IDT PrimerQuest, Coralville, IA, USA). Prime sequences are mouse β-actin-forward GGCTGTATTCCCCTCCATC, mouse β-actin-reverse ATGCCATGTTCAATGGGGTA, mouse TNF-α-forward ACCACGCTCTTCTGTCTA, mouse TNF-α-reverse GAAGATGATCTGAGTGTGAGG, mouse NOX-2-forward TCCAGTGCGTGTTGCTCGACAA, mouse NOX-2-reverse ATTGTGTGGATGGCGGTGTGCA, mouse CCL4-forward TGCTCGTGGCTGCCTTCTGT, mouse CCL4-reverse TGTGAAGCTGCCGGGAGGTGTA, mouse CCL2-forward ACCACCATGCAGGTCCCTGTCAT, mouse CCL2-reverse AGCCAACACGTGGATGCTCCAG, mouse IL-1β-forward CTCTTGTTG ATGTGCTGCTG, mouse IL-1β-reverse GAC TG TTCTTTGAAGTTGACG, human CD52-forward AGCCCTGAGATCACCTAAA, and human CD52-reverse GAGTCCAGTTTGTATCTGTACC. Settings for analysis using a C-1000 CFX96 machine (Bio-Rad, Hercules, CA, USA) were as follows: initial denaturation (95 °C/2 min) was followed by 45 cycles of denaturation (95 °C/10 s) and primer annealing and elongation (60 °C/30 s). A melt curve was performed on all samples for quality control. Data was quantified by the 2(−ΔΔCt) method using β-actin as an internal control reference mRNA. If the Ct values for β-actin reference mRNA were more than 2 cycles higher than the average, those samples were considered poor quality cDNA and omitted from analysis. Data analysis was performed using CFX Maestro software version 2.3 provided by BioRad (Hercules, CA, USA).

### 2.9. Flow Cytometry

Cells were harvested, counted, washed twice, and stained with mAbs, as indicated, in flow buffer (PBS with 1% heat-inactivated FBS). Cells were stained with mAbs at 4 °C in the dark for 20 min, washed, and put in flow buffer for analysis. Cells were gated on live cells using FSC and SSC. Flow cytometry antibodies (Biolegend, San Diego, CA, USA): human CD4-FITC (1/100 dilution, 317408), human CD8-PE (1/100, 300908), human CD3-FITC (1/100, 317306), human CD127-FITC (1/100, 351312), mouse CD19-PE (1/100, 115508), human CD39-PE (1/100, 328208), human VLA4-PE (1/100, 304303), human HLA-DR-FITC (1/50, 307604). CAR expression was assessed using biotin-Protein L (1 μg/mL, GenScript M00097) with APC (405207)- or PE (405203)-streptavidin (1/2000, Biolegend). Intracellular staining using anti-FoxP3-APC (1/100, Invitrogen 17-4776-42) and isotype-APC (1/200, Invitrogen 17-4321-81) followed membrane permeabilization using eBioscience™ Foxp3/Transcription Factor Staining Buffer Set (Invitrogen 00-5523-00, Waltham, MA, USA). Cells were analyzed by staining with a C6 Accuri (BD Biosciences, Franklin Lakes, NJ, USA) flow cytometer or an 8-color MacsQuant (Miltenyi Biotech, Bergisch Gladbach, Germany).

### 2.10. CAR Antigen Activations and Enzyme-Linked Immunosorbent Assays

Plate-bound presentation of recombinant G93A hSOD1 was performed in ELISA plates (Corning 3361) that were coated for 2 h with 50 μL/well of G93A hSOD1 (0.2 or 2 μg/mL; provide by R. Roos Lab, University of Chicago) and then rinsed with PBS. Transduced Tregs were added to the ELISA plate wells at 50,000 cells/0.2 mL/well in XVIVO-15 with 10% human sera and incubated at 37 °C for 24 h. Cell-free media was collected and measured for IL-10 (430604) and BDNF (446604) according to the manufacturer’s instructions (Biolegend).

### 2.11. Statistical Analysis

The statistical evaluation was performed using GraphPad Prism 9 (GraphPad, San Diego, DA, USA) using a one-way ANOVA followed by Dunnett’s multiple comparisons test for comparison of more than two experimental groups. An unpaired *t*-test with Welch’s correction was used to determine a significant difference between two groups of data. The Gehan–Breslow–Wilcoxon test was used for Kaplan–Meier curve analysis. A *p* value of less than 0.05 was considered significant.

## 3. Results

### 3.1. The Anti-SOD1 scFv Binds to Spinal Cord from Most ALS Patients

**Figure 1:**
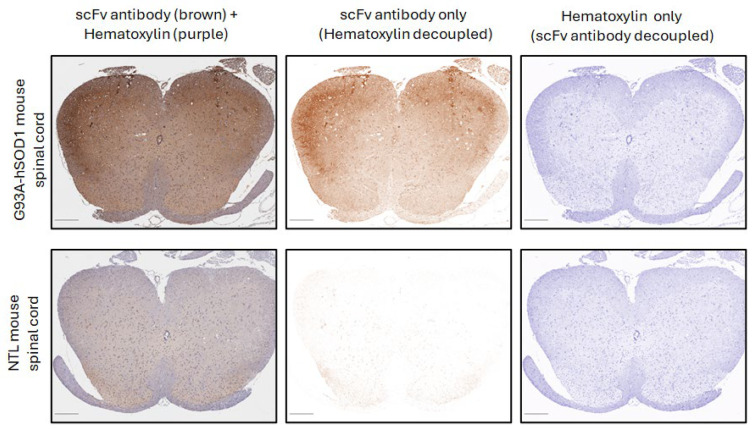
Immunohistochemical staining of mouse spinal cord tissue from G93A hSOD1 with an scFv antibody composed of anti-misfolded hSOD1 CAR scFv fused to a rabbit Fc. The left panels show scFv antibody staining (brown) and hematoxylin counterstain (purple) together. The middle panels show the scFv antibody decoupled from the hematoxylin counterstain. The right panels show the hematoxylin counterstain decoupled from the scFv antibody staining. The top panels are a spinal cord cross-section from an mSOD1-NSG mouse. The bottom panels show a spinal cord cross-section from an age-matched non-transgenic littermate (NTL). Scale bars in the bottom left corner of panels represent 250 μm.

**Figure 2:**
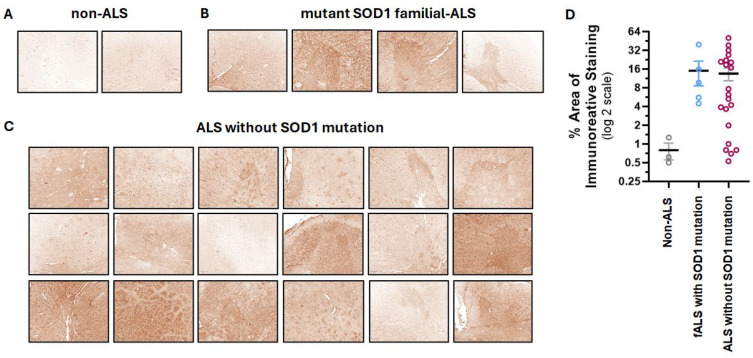
Immunohistochemical staining of human spinal cord tissue from postmortem ALS patients with the anti-hSOD1 CAR scFv fused to a rabbit Fc. Representative staining images of the scFv-Fc staining (brown) decoupled from the hematoxylin counterstain in tissues from non-ALS controls (**A**), fALS patients with a SOD1 mutation (**B**), and ALS patients with no SOD1 mutation (**C**). Scale bars in the bottom left corner of panels represent 250 μm. Quantification of the extent of DAB-labeled scFv antibody staining in tissues from non-ALS (*n* = 3), fALS with SOD1 mutation (*n* = 5), and ALS patients without SOD1 mutations (*n* = 21) cases (**D**). Circles are individual case samples, black horizontal lines are means, and colored bars are SEMs.

To target the disease sites in ALS patients, we have developed a CAR based on the human 16L-40 scFv clone that binds preferentially to misfolded human SOD1 over wild-type SOD1. This scFv binds to a site that is exposed upon misfolding of SOD1 that is 50 amino acids away from the G93A mutation, and it has been shown to bind preferentially to SOD1 with mutations at G93A, G85R, or A4V [31]. We created an scFv antibody using this fully human scFv attached to a rabbit Fc and confirmed that indeed this scFv preferentially binds mutant hSOD1 relative to WT hSOD1 as shown by indirect ELISA [17]. To understand the potential of this scFv to bind ALS-relevant misfolded SOD1 in CNS tissue, we tested this misfolded hSOD1 scFv antibody for binding by IHC in spinal cord samples from transgenic NSG mice expressing G93A hSOD1 (mSOD1-NSG) and non-transgenic littermates (NTL). We observed prominent staining in the ventral horn area of the spinal cord from mSOD1-NSG transgenic but not NTL (Figure 1). The presence of misfolded SOD1 in ALS patients is controversial. Scientists report that misfolded SOD1 is also found in most ALS patients, even if they do not have a genetic mutation in SOD1 [9,10,11]. While other researchers claim that the misfolded protein is only in fALS patients who have a SOD1 mutation [12]. It is important to note that different misfolded SOD1 antibodies and IHC protocols were used among these disparate groups. We avoided the use of EDTA or any other metal chelators in our IHC protocol that could prevent observing differences between normal and misfolded SOD1, and we found that an antigen retrieval step was not required. We tested our misfolded hSOD1-binding scFv-Fc antibody in spinal cord anterior horn regions from ALS patients with (*n* = 5) and without (*n* = 21) SOD1 mutations. All patients with known SOD1 mutations stained with the scFv antibody with greater intensity than the non-ALS control spinal cord samples, and 16 ALS patients with no SOD1 mutations stained similarly to the fALS patients with SOD1 mutations (Figure 2). There was a range of staining intensity from the samples taken from ALS patients without SOD1 mutations: some had higher intensity similar to those from ALS patients with known SOD1 mutations, some had a lower amount of staining, and five samples (24%) were similar to non-ALS controls. Overall, 76% of ALS patient samples showed staining for misfolded SOD1 above background using the 16L-40 scFv-Fc protein.

### 3.2. Co-Expression of BDNF and Anti-Misfolded Human SOD1 CAR in Human Tregs

**Figure 3:**
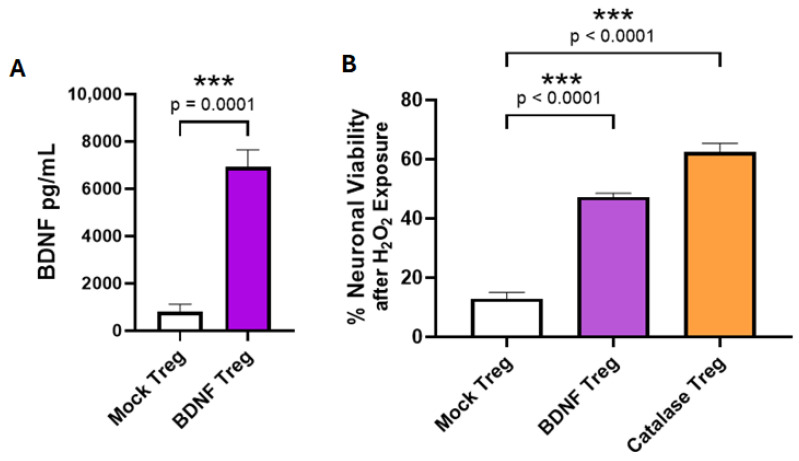
Human Tregs expanded in vitro for 17 days and transduced with a BDNF retroviral vector secrete BDNF and are neuroprotective. (**A**) BDNF Tregs secreted BDNF into culture media, whereas little BDNF was measured in media of mock Tregs (*n* = 6, mean ± SEM, *p* value determined by unpaired *t*-test with Welch’s correction). (**B**). The viability of human neuronal cells co-cultured with Tregs in the presence of H_2_O_2_. Data are shown as % viability compared to untreated Luc-SH-SY5Y neuronal cells. Tregs transduced with BDNF or catalase inhibited H_2_O_2_-induced Luc-SH-SY5Y loss relative to mock-transduced Tregs (*n* = 4, mean ± SEM, *p* value determined by Dunnett’s multiple comparisons test versus mock Treg). *** indicates *p* < 0.001.

We previously demonstrated that the anti-SOD1 CAR with a CD28 costimulatory domain and a CD3ζ cytoplasmic signaling domain expressed in human Tregs elicited an immunomodulatory effector response after reacting with aggregated misfolded hSOD1 [17]. To create an enhanced neuroprotective Treg-based cell therapy for ALS, we chose to add therapeutic activity to Tregs by having them constitutively express a disease-modifying molecule (DMM). BDNF is known to have protective effects for neuron survival, improvements in memory, and other neurological functions. As shown in Figure 3A, BDNF-transduced Tregs secreted BDNF in cell culture, whereas mock-transduced Tregs did not. To evaluate whether the Treg-produced BDNF retains neuroprotective activity, we tested the BDNF-transduced Tregs for their ability to inhibit the death of neuronal cells due to peroxidative stress in vitro. The SH-SY5Y human neuronal cell line was exposed to 80 μM H_2_O_2_ in the presence of co-cultured BDNF-transduced Tregs that expressed BDNF constitutively, positive control Tregs that were transduced to express catalase, or mock-transduced Tregs. Catalase is an enzyme that directly inactivates H_2_O_2_. The Tregs that produced BDNF or catalase were able to prevent peroxidase-induced death of the neuronal cells compared to neuronal cells co-cultured with mock Tregs (Figure 3B).

**Figure 4:**
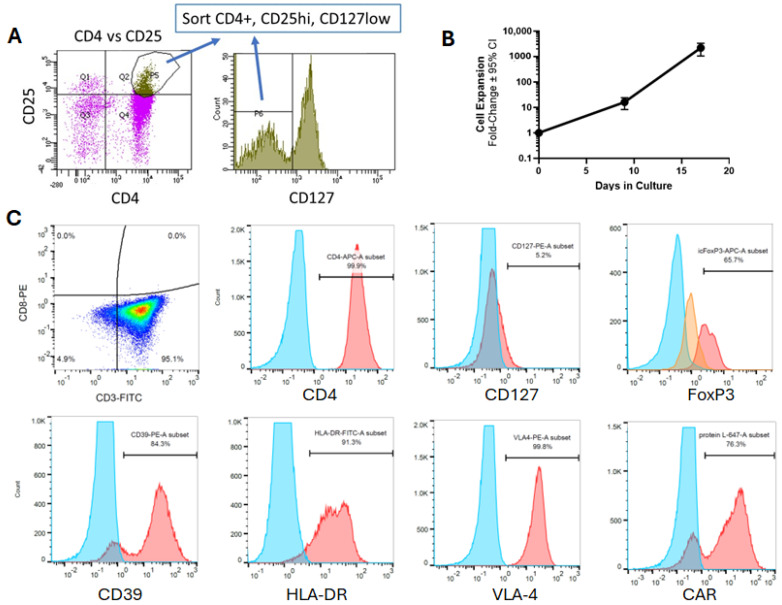
Characteristics of human Tregs transduced with a retroviral vector expressing both a CAR and BDNF. (**A**) Representative flow cytometry plots of the isolation of Tregs from human PBMCs and sorted for CD4+CD25hiCD127lo cells. Q1, Q2, Q3, and Q4 refer to quadrants. (**B**) Treg expansion over 17 days in vitro. Values are mean fold-change ± SEM relative to day 0 from twelve independent Tregs sorts from ten different human donors. (**C**) Representative flow cytometry plots of CAR- and BDNF-transduced Tregs after 17 days of expansion for different cell markers and CAR expression. Red is specific staining, and blue is unstained control. Orange is isotype control for intracellular FoxP3 staining.

Since BDNF-transduced Tregs demonstrated neuroprotective activity, we generated a retroviral construct containing the anti-SOD1 CAR combined with human BDNF separated by a T2A peptide for co-expression in Tregs. Tregs sorted from human PBMCs were >97% CD4+CD25hiCD127lo cells (Figure 4A). Upon stimulating these sorted Tregs on days 0 and 9 in vitro, and transducing them with the combined CAR and BDNF vector on days 10 and 11, these Tregs could be expanded over 1000-fold by day 17 in vitro (Figure 4B). These expanded CAR and BDNF Tregs maintained the CD4+CD127lo phenotype and expressed other known markers of human Tregs, including surface markers MHC class II, CD39, and VLA4, and intracellular FoxP3 (Figure 4C). Furthermore, these Tregs expressed CAR.

**Figure 5:**
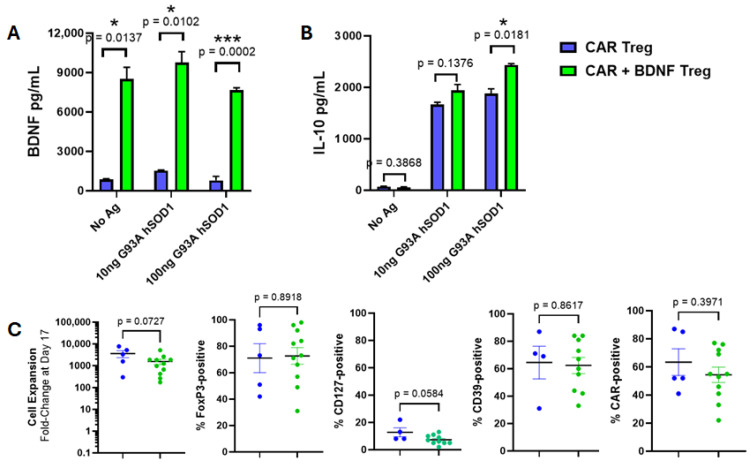
Human Tregs expressing both CAR and BDNF are similar to Tregs expressing CAR alone other than constitutive secretion of BDNF. (**A**) CAR + BDNF Tregs secreted BDNF into culture media in wells coated with G93A hSOD1 or media only. Little BDNF was measured in media of CAR Tregs (*n* = 3 mean ± SEM, *p* value determined by unpaired *t*-test with Welch’s correction). (**B**) CAR + BDNF Tregs and CAR Tregs secreted IL-10 upon culture on G93A hSOD1-coated wells, whereas no IL-10 was measured in the absence of CAR antigen (*n* = 3 mean ± SEM, *p* value determined by unpaired *t*-test with Welch’s correction). (**C**) CAR + BDNF Tregs (green) and CAR Tregs (blue) have similar levels of cell expansion, expression of FoxP3, CD127, CD39, and CAR as measured by flow cytometry (Each circle is an individual experiment, for cell expansion, %FoxP3, and %CAR *n* = 5 for CAR Treg and *n* = 11 for CAR and BDNF Tregs, for %CD127 and %CD39 *n* = 4 for CAR Treg and *n* = 10 for CAR and BDNF Tregs, black horizontal lines are means, and colored bars are SEMs, *p* value determined by unpaired *t*-test with Welch’s correction). * *p* < 0.05; *** *p* < 0.001.

Tregs expressing CAR alone were compared in vitro to Tregs co-expressing CAR and BDNF. CAR-alone Tregs produced low amounts of BDNF, whereas Tregs co-expressing both CAR and BDNF secreted high amounts of BDNF, whether CAR was activated against a human SOD1 antigen or not (Figure 5A). These CAR and BDNF Tregs were similar to Tregs expressing CAR alone. That is, co-expression of BDNF did not attenuate CAR-mediated IL-10 production when activated by specific antigen (Figure 5B). Treg expansion in vitro, Treg phenotype markers, and CAR expression levels were also unchanged in CAR alone and in BDNF co-expressed Tregs (Figure 5C).

### 3.3. Human Tregs Expressing Both CAR and BDNF Delay Disease and Inhibit Neuroinflammation in mSOD1-NSG Mice

Human Treg therapies cannot be tested in immune-intact mouse models without being rejected. Mouse Treg properties, expansion in vitro, and transduction strategies are very different from human Tregs. Our CAR scFv is also of human origin and may be immunogenic in mice. Taken together, an ALS mouse model that permits the testing of human CAR Treg therapies would be ideal for in vivo testing. We developed a mouse model of ALS using NSG mice expressing G93A human SOD1 that develops ALS-like motor neuron disease with spinal cord neuroinflammation while permitting long-term survival of human cells due to the absence of a murine adaptive immune response [27].

**Figure 6:**
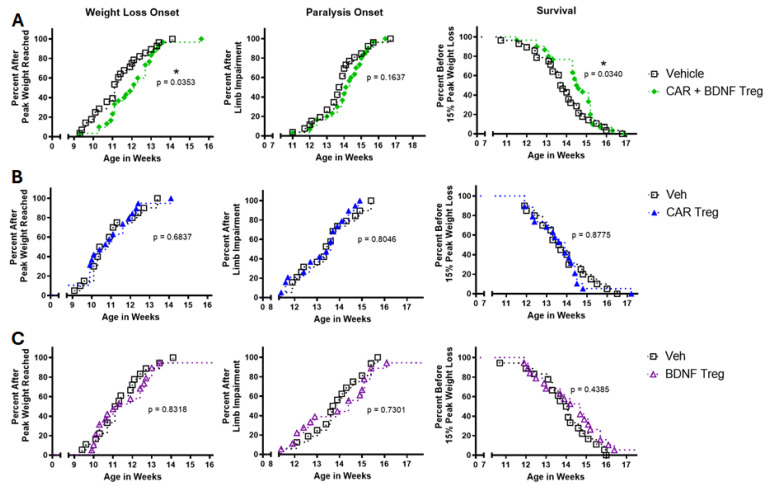
CAR and BDNF Tregs delay disease and increase survival of mSOD1-NSG mice. Human Tregs expressing CAR and BDNF, CAR alone, or BDNF alone were injected into mSOD1-NSG mice at 7.5 weeks of age. (**A**) CAR + BDNF Treg-injected mice had a delay in weight loss onset (*p* = 0.035) and increased survival (*p* = 0.034), but no change in onset of paralysis (*p* = 0.164), relative to vehicle-injected mice. Kaplan–Meier curve consists of *n* = 30 CAR + BDNF Treg-treated mice and *n* = 28 vehicle-treated mice. (**B**) CAR-alone Treg-injected mice had no delay in weight loss onset (*p* = 0.684), onset of paralysis (*p* = 0.805), or change in survival (*p* = 0.878). Kaplan–Meier curve consists of *n* = 19 CAR Treg-treated mice and *n* = 20 vehicle-treated mice. (**C**) BDNF-alone Treg-injected mice had no delay in weight loss onset (*p* = 0.832), onset of paralysis (*p* = 0.730), or change in survival (*p* = 0.439). Kaplan–Meier curve consists of *n* = 19 BDNF Treg-treated mice and *n* = 18 vehicle-treated mice. Gehan–Breslow–Wilcoxon test was used to determine *p* values. * *p* < 0.05.

CAR and BDNF human Tregs or vehicle were injected IV into mSOD1-NSG mice at 8 weeks of age. Mice were IP injected with IL-2 on days 2, 4, and 6 to support initial Treg survival. Mice injected with CAR and BDNF Tregs delayed weight loss onset and survival but did not change the onset of paralysis (Figure 6A). Mice injected with CAR-only Tregs or BDNF-only Tregs had no effect compared to vehicle-injected mice (Figure 6B,C), indicating that the CAR and BDNF combination was important for in vivo activity.

**Figure 7:**
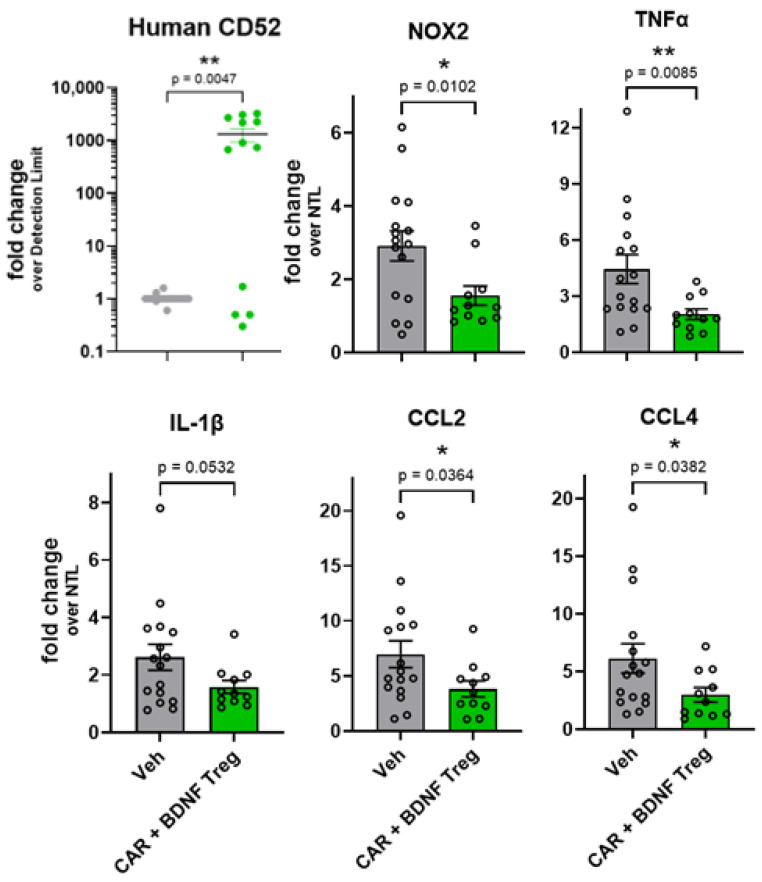
CAR and BDNF Tregs decrease inflammation in the spinal cord of mSOD1-NSG mice. Spinal cord tissue from G93A-hSOD1 transgenic NSG mice at 13 weeks of age was evaluated for mRNA expression of inflammatory mediators. Human Tregs expressing CAR and BDNF (green) or vehicle (gray) were injected at 8 weeks of age. RNA from spinal cord was isolated. Human CD52 mRNA, an indicator of human Tregs, was found in 8 of 12 mice injected with CAR and BDNF Tregs at 13 weeks. Mouse NOX-2, TNF-α, IL-1β, CCL2, and CCL4 mRNAs were decreased in CAR + BDNF Treg-injected mice relative to vehicle-injected mice. Circles represent individual mice (*n* = 12 CAR + BDNF and *n* = 16 vehicle) combined from three experiments. Mouse mRNA values are expressed as fold-change over values from age-matched non-transgenic littermate controls (NTLs, *n* = 12). *p* values determined by unpaired *t*-test with Welch’s correction. * *p* < 0.05; ** *p* < 0.01.

To assess neuroinflammation in the spinal cord, CAR and BDNF Tregs or vehicle were injected into mSOD1-NSG mice at 8 weeks of age, and spinal cords were isolated at 13 weeks to assess neuroinflammatory markers. To determine the presence of human Tregs, human CD52 mRNA was also measured in the mouse spinal cords because this gene is highly expressed in human Tregs, and the mouse does not express CD52. Human CD52 was detected in the spinal cords of 67% of mice injected with Tregs at 13 weeks (Figure 7). Expression of NOX-2, TNF-α, CCL2, and CCL4 is upregulated in the spinal cord with age in transgenic mice expressing G93A hSOD1 [14,27], and Tregs have been shown to decrease their expression [14,16,17]. Mouse NOX-2, TNF-α, CCL2, and CCL4 mRNAs were decreased in mice injected with CAR and BDNF Tregs, while there was a trend for decreased IL-1β compared to vehicle-injected mice (Figure 7).

## 4. Discussion

Cellular therapy for cancers using CAR T cells to target and kill tumor cells has emerged as a highly effective treatment, leading to seven FDA-approved therapies to date. CAR T cells are being pursued in some autoantibody-mediated autoimmunity diseases, such as multiple sclerosis and rheumatic diseases, by targeting and killing B cells [32]. Tregs, on the other hand, are considered a promising therapeutic strategy for diseases such as diabetes, organ transplant rejection, and neurodegeneration. Several neurodegenerative diseases, including ALS, have misfolded protein aggregates, neuroinflammation, and signs of oxidative/nitrosative damage in the areas of progressive neuron loss. Tregs are associated with slower disease development in ALS [13,14] and ALS animal models [14,15]. In addition, several strategies to increase the number of polyclonal Tregs are being tested in ALS clinical trials [24,25,26]. In this study, we describe a genetically engineered enhanced Treg that targets the motor neuron regions and delivers its inherent anti-inflammatory activity and an additional neuroprotective disease-modifying molecule.

We demonstrate the feasibility of using targeted CAR Tregs as a potential therapy for human ALS. It remains unclear whether having CAR target glial cells, neurons, or disease-specific markers will be the most effective approach. We have focused on misfolded and aggregated SOD1 for directing Tregs to deliver disease-modifying activity at disease sites within the CNS, minimizing targeting effects in other CNS regions.

ALS is a complex heterogenous disease; it is likely that multiple mechanisms of action will be required to modify the disease course in patients. To address this, we have added DMMs as cargo genes to be expressed by CAR Tregs. BDNF is a lead candidate DMM because BDNF is known to have protective effects for neuron survival, improvements in memory, and affects other neurological functions. BDNF has been tested directly in ALS patients [33,34]. Although injections of BDNF protein alone did not show a benefit, there were individuals in the study who reported benefits [35]. BDNF is also being pursued in other neurodegenerative diseases [36]. We demonstrated that CAR Tregs expressing BDNF protected a neuronal cell line from peroxidative-induced death in vitro.

In a previous study, we demonstrated that using a scFv that binds to the misfolded SOD1 better than to wild-type SOD1 leads to the recognition of aggregated SOD1 within the CNS tissue explant assay from an ALS mouse model [17]. As the disease progresses, misfolded SOD1 aggregates are found sufficiently outside of cells in the CNS to be recognized by a CAR Treg. This recognition did not occur in other tissues (lung and liver), even though all cells express the same G93A SOD1 transgene.

In the ALS field, there is an ongoing debate regarding the presence of misfolded SOD1 aggregates in the CNS of patients with ALS who do not have a mutation in SOD1. While some studies suggest widespread expression of SOD1 aggregates in these patients [9,10,11], others report that only individuals with SOD1 mutations exhibit such aggregates [12]. Our findings align with the former, demonstrating that approximately 76% of ALS patients could be identified using an antibody that selectively binds misfolded SOD1 rather than wild-type SOD1.

Animal models containing SOD1 mutations associated with ALS provide a platform to evaluate therapeutic activity in vivo. However, these models have limitations and have failed to predict clinical outcomes accurately [37]. To improve predictability, they must be employed cautiously to mitigate experimental bias. Their primary utility lies in pharmacological studies, where they can reveal drug pharmacokinetics and activity at the disease site. There are numerous animal models, including those involving mice, rats, and larger animals, that incorporate SOD1 mutations to investigate ALS [38]. The progression of disease varies based on the specific mutation and the genetic background of the strain [38,39,40]. The most commonly studied transgenic human SOD1 mutation, G93A, leads to rapid disease progression. We have introduced the G93A SOD1 mutation into the NSG mouse background, enabling the transplantation of human cells [27]. This mSOD1-NSG model exhibits an aggressive disease course, with onset occurring around 77 days and an endpoint at 98 days. This creates a narrow window for assessing disease-modifying interventions.

In the current study, we went further and were able to show that human Tregs injected IV were able to reach the CNS, modify proinflammatory gene expression, delay disease onset, and slightly delay survival. BDNF was an important component of the in vivo efficacy of CAR Tregs in the mSOD1-NSG ALS model. While the neuroprotective properties of BDNF are well known, this neurotrophic factor has also been described to have immunomodulatory activity by enhancing IL-10 and inhibiting TNF-α in the CNS [41,42]. Having dual activities of neuroprotection and anti-inflammation makes BDNF an attractive disease-modifying molecule for CAR Tregs to express. This in vivo proof of concept shows that the CAR Treg concept has the potential to be beneficial for ALS therapy.

The NSG background enabled us to test the actual human therapeutic cell. However, this model has certain limitations, including an imperfect match between human cells and their murine counterparts (e.g., adhesion molecules and cytokines), the absence of a complete immune system, a lack of responsiveness to IL-4, and no endogenous source of IL-2. Consequently, it is reasonable to expect that human CAR Tregs may not be as effective in modifying the disease process in mice as they might be in human patients. For this reason, we primarily regard this model as a pharmacological tool to evaluate Treg persistence, activity, and localization, rather than a model specifically designed to assess disease course modification.

Relative to conventional T cells in traditional CAR T cell therapies, Tregs represent a much smaller population and expand more slowly in vitro. In this current work, we show that Treg expansion was typically over 200-fold within 2.5 weeks, and it is reasonable to generate more than one billion CAR Tregs from a patient’s apheresis. This is more than a sufficient cell dose for treatment in ALS patients and comparable to doses used with polyclonal Tregs [24,25,26].

Our data did show that Tregs were able to modify disease activity when both a CAR to target the Tregs and the co-expression of BDNF were used, compared to either alone. These data demonstrate that for an aggressive CNS disease that Treg activity alone may be insufficient and that additional therapeutic modalities may be needed. This highlights an advantage of a cell and gene therapy-based Treg strategy. CAR Tregs can be modified to produce a number of different DMMs that can alter different pharmacological pathways. We have demonstrated a role for BDNF in this study, but further improvements in Treg effector functions, increased persistence, and CNS penetration may lead to even greater therapeutic benefits.

## Data Availability

The authors state that all data supporting the conclusions of this study are included in the article. Additional raw and processed data can be provided by the corresponding author upon reasonable request.

## Abbreviations

The following abbreviations are used in this manuscript:

ALS	amyotrophic lateral sclerosis
fALS	familial ALS
sALS	sporadic ALS
CNS	central nervous system
Tregs	regulatory T cells
DMM	disease-modifying molecule
BDNF	brain-derived neurotrophic factor
scFv	single-chain variable fragment
CAR	chimeric antigen receptor
SOD1	superoxide dismutase-1
hSOD1	human SOD1
NSG	NOD.Cg-Prkdc scid IL2rγ tm1Wjl
mSOD1-NSG	G93A hSOD1-NSG transgenic mice
NTLs	non-transgenic littermates
Luc-SH-SY5Y	luciferase-expressing SH-SY5Y cell line
IHC	immunohistochemistry
PBMCs	peripheral blood mononuclear cells
IP	intraperitoneal
IV	intravenous
